# LncRNA RCAT1 promotes tumor progression and metastasis via miR-214-5p/E2F2 axis in renal cell carcinoma

**DOI:** 10.1038/s41419-021-03955-7

**Published:** 2021-07-09

**Authors:** Renbo Guo, Benkui Zou, Yiran Liang, Jiasheng Bian, Jian Xu, Qian Zhou, Chao Zhang, Tao Chen, Mingshan Yang, Huansheng Wang, Fajun Pei, Zhonghua Xu

**Affiliations:** 1 Department of Urology, Qilu Hospital, Cheeloo College of Medicine, Shandong University, Jinan, Shandong China; 2 Department of Urology, Shandong Cancer Hospital and Institute, Shandong First Medical University and Shandong Academy of Medical Sciences, Jinan, Shandong China; 3 Department of Breast Surgery, Qilu Hospital, Shandong University, Jinan, Shandong China

**Keywords:** Cancer, Molecular biology

## Abstract

Renal cell carcinoma is the second malignant tumors in the urinary system with high mortality and morbidity. Increasing evidence suggests that long non-coding RNAs (lncRNAs) play critical roles in tumor development and progression. In the current study, based on the publicly available data obtained from GEO and TCGA database, we identified five prognosis-related lncRNAs with the ability to predict the prognosis of patients with renal cell carcinoma. Among them, the uncharacterized and upregulated lncRNA RCAT1 (renal cancer-associated transcript 1) was identified as the key lncRNA. Our data further revealed that the expression of lncRNA RCAT1 was significantly upregulated in renal cell carcinoma tissues and cells. Gain-of-function and loss-of-function studies showed that lncRNA RCAT1 promoted cell proliferation, migration, and invasion in vitro and in vivo. Furthermore, we verified that lncRNA RCAT1 could abundantly sponge miR-214-5p, which served as a tumor suppressor in renal cell carcinoma. Significantly, miR-214-5p overexpression could attenuate the promotion of cell proliferation and metastasis induced by lncRNA RCAT1. Moreover, we found that E2F2 was a direct target of miR-214-5p, and lncRNA RCAT1 could protect E2F2 from miR-214-5p-mediated degradation. Taken together, our findings suggested that lncRNA RCAT1 could enhance the malignant phenotype of renal cell carcinoma cells by modulating miR‐214‐5p/E2F2 axis, and lncRNA RCAT1 might be a novel prognostic biomarker and a potential therapeutic target for renal cell carcinoma.

## Introduction

Clear cell renal cell carcinoma (ccRCC) is the predominant pathological subtype of renal cell carcinoma and accounts for 80∼90% of all renal cancers in adults [1]. Since the disease course of ccRCC is usually asymptomatic and there are no effective early diagnostic markers, approximately 30% of ccRCC patients have occurred distant metastasis and 40% of ccRCC patients have already occurred local relapse at the time of their initial diagnosis [2]. Moreover, metastasis and recurrence significantly hinder treatment success and lead to dramatically reduced overall survival (OS) rate of ccRCC patients [3]. Therefore, gaining insight into the underlying mechanisms of ccRCC progression is particularly important for identifying effective biomarkers and therapeutic targets to improve the diagnosis and prognosis of ccRCC patients.

Long non-coding RNAs (lncRNAs) are a novel class of non-coding RNAs longer than 200 nucleotides in length with limited or no protein-coding capacity [4]. Accumulating studies demonstrate that lncRNAs participate in multiple biological processes, through serving as oncogenes or tumor-suppressor, such as cell proliferation, apoptosis, metastasis, and cell differentiation [5]. For example, EGFR-AS1 promotes RCC cell growth and metastasis by interacting with HuR to increase the mRNA stability of EGFR [6]. Moreover, lncRNA FILNC1 plays a critical role in the energy metabolism and development of renal cancer through interacting with AUF1 to downregulate the expression of c-Myc, and low FILNC1 expression is associated with poor clinical outcomes [7]. These studies indicated that lncRNAs might serve as a potential biomarker for the diagnosis and prognostic prediction in RCC.

Various studies reported that lncRNAs could serve as competing endogenous RNAs (ceRNAs) to compete for miRNA response elements (MREs) with mRNAs [8], thus modulating the expression of miRNA targets. lncRNA CDKN2B-AS1 could block miR-141-mediated cyclin D suppression to enhance tumor progression and metastasis [9]. HOXA11‐AS sponges miR‐146b‐5p to upregulate MMP16 expression and renal cancer progression [10]. However, more efforts are needed to reveal the functional roles and exact mechanisms of numerous lncRNAs in ccRCC.

Using bioinformatics analysis, we identified a novel lncRNA RCAT1 (renal cancer-associated transcript 1) as a significant tumor promoter in RCC. The expression of lncRNA RCAT1 was significantly upregulated in RCC tissues and associated with poor prognosis of RCC patients. Further study revealed that lncRNA RCAT1 could serve as the miR-214-5p sponge to promote ccRCC cell proliferation and migration. Our study could provide a better understanding about the ccRCC progression and a promising biomarker for prognosis prediction and treatment for ccRCC patients.

## Results

### Identification of differentially expressed lncRNAs in ccRCC and normal tissues

**Fig. 1:**
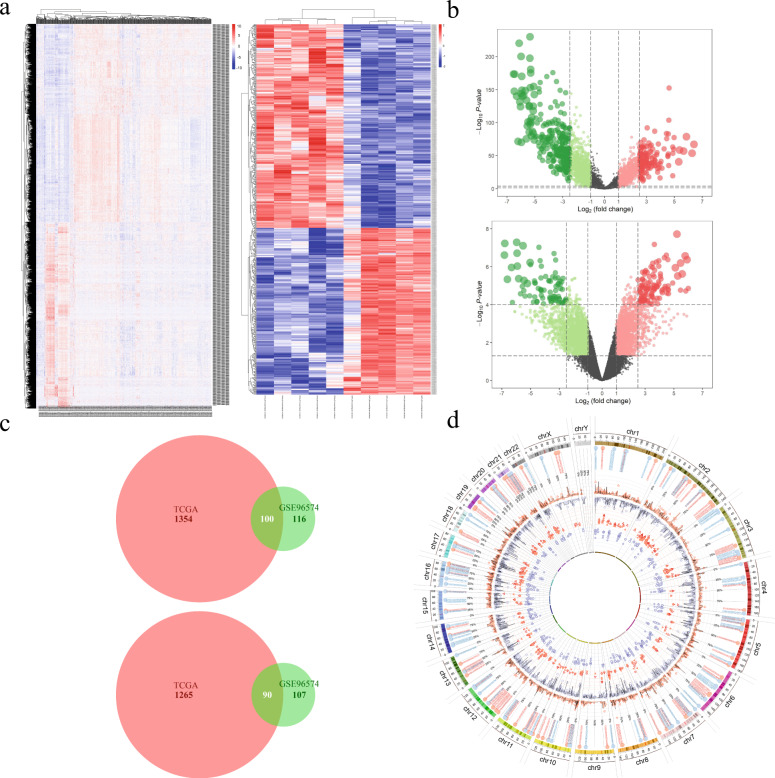
Identification of differentially expressed lncRNAs. **a**, **b** Hierarchical clustering heatmap (**a**) and volcano plots (**b**) of TCGA and GSE96574 showing the differentially expressed lncRNAs between ccRCC samples and normal samples. **c** Comparison of the differentially upregulated or downregulated lncRNAs in the two datasets. **d** A Circos plot was used to show the differentially expressed lncRNAs (log_2_ fold change (FC) | > 1, *P* < 0.05). The inner circle shows the downregulated lncRNAs (purple dots) and the second circle identifies the upregulated lncRNAs in ccRCC tissues (red triangles) according to GSE96574. The third circle shows the downregulated lncRNAs (purple dots) and the fourth circle identifies the upregulated lncRNAs in ccRCC tissues (red triangles) according to TCGA. The outside circle represents the overlap of upregulated (pink dot) or downregulated lncRNAs (blue dots).

Based on integrated analysis of the lncRNA-expression profiles obtained from TCGA and GEO datasets, we identified 2809 (1454 upregulated and 1355 downregulated lncRNAs) and 413 (216 upregulated and 197 downregulated lncRNAs) differentially expressed lncRNAs (DElncRNAs), respectively (Fig. 1a, b). After taking the intersection of DElncRNAs of the two datasets, 100 commonly upregulated lncRNAs and 90 commonly downregulated lncRNAs were obtained (Fig. 1c, d).

### Construction of five-lncRNAs-based prognostic model

**Fig. 2:**
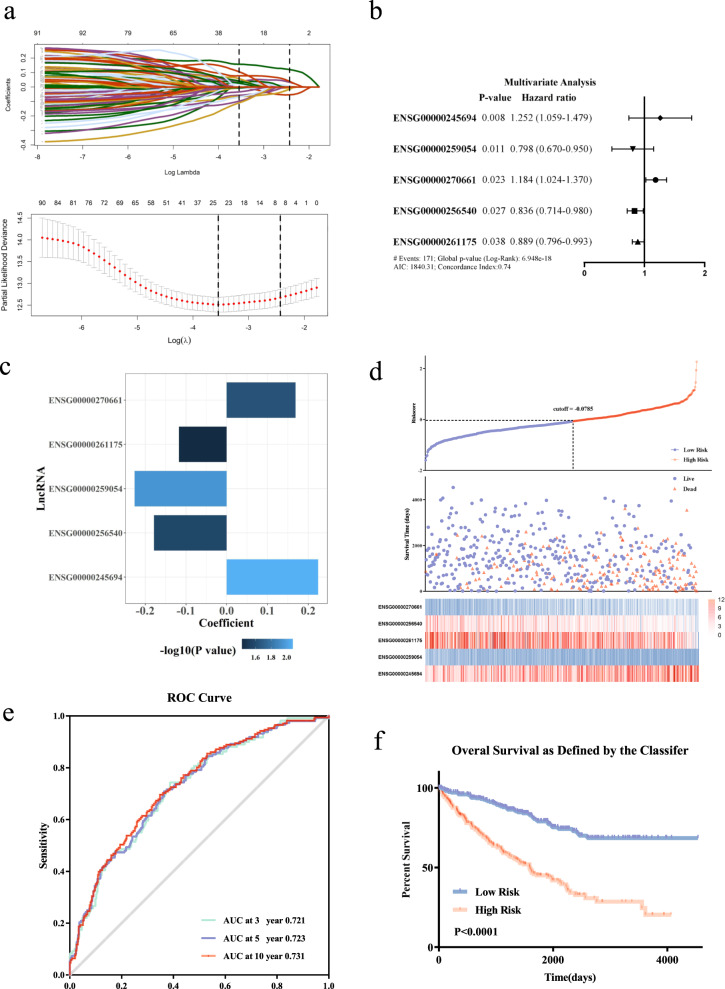
The lncRNA prognostic model. **a** Lasso regression analysis of 94 DElncRNAs. Those with *P* < 0.05 were showed. **b** A forest plot illustrating the HR and *P*-value from the multivariate cox regression analysis of 24 DElncRNAs. Those with *P* < 0.05 were showed. **c** The bar plot shows coefficients of five lncRNAs in the prognostic model. **d** Risk score system of the prognostic model. The above scatterplot exhibits the risk scores of each ccRCC patient with survival data. The middle scatterplot showed the relationship between the risk scores and the survival status/survival time. The below heatmap displays the expression profiles of the five lncRNAs in the prognostic model. **e** The time-dependent ROC curve of OS suggests the reliability of the prognostic model. **f** Kaplan–Meier overall survival curves for ccRCC patients exhibited that the OS of high-risk group was shorter than the OS of low-risk group.

To further investigate whether the above DElncRNAs were closely associated with the OS of ccRCC patients in the TCGA cohort, we firstly carried out univariate Cox regression analysis. The results indicated that 94 lncRNAs were significantly related with the OS of ccRCC patients (Table S1). Lasso analysis was performed and 24 lncRNAs were further selected (Fig. 2a). Subsequently, multivariate Cox regression analysis was utilized to find the independent risk factors for OS. Five lncRNAs were finally selected and their regression coefficients were calculated based on multivariate Cox regression analysis (Fig. 2b). A prognostic model based on the expression levels and regression coefficients of each lncRNA was further constructed in TCGA cohort (Fig. 2c and Table S2). The risk score for each patient was calculated according to the following formula: risk score = (0.169 × expression level of ENSG00000270661) + (−0.179 × expression level of ENSG00000256540) + (−0.117 × expression level of ENSG00000261175) + (−0.226 × expression level of ENSG00000259054) + (0.224 × expression level of ENSG00000245694). Furthermore, using the optimal cutoff value of risk scores, patients were classified into the high‐risk group (*n* = 241) and low‐risk group (*n* = 281). The survival rate and survival time of ccRCC patients in the high-risk group were significantly decreased compared to the low-risk group (Fig. 2d). The time-dependent receiver operating characteristic (ROC) curves indicated that the area under the ROC (AUC) for OS was 0.721 at 3 years, 0.723 at 5 years, and 0.731 at 10 years (Fig. 2e). The Kaplan–Meier survival curve revealed that high-risk group was closely associated with shorter OS compared to the low-risk group (Fig. 2f), indicating that the prognostic model performed satisfactorily to predict prognosis in ccRCC patients.

### Construction of the ceRNA Network

In order to investigate the potential mechanism of the five DElncRNAs, we constructed a network based on the ceRNA theory. The DEmiRNAs and DEmRNAs were screened using TCGA databases. Compared with normal samples, 109 DEmiRNAs (42 upregulated and 67 downregulated miRNAs) and 5100 DEmRNAs (2115 upregulated and 2985 downregulated miRNAs) were obtained (Fig. S1a, b). DIANA-LncBase v2 was used to predict the interaction between lncRNAs and miRNAs. After crosschecking with the DEmiRNAs, only 34 DEmiRNAs were found to be associated with four lncRNAs (Fig. S1c). The interaction between miRNA and mRNA was predicted using miRDB, miRTarBase, TargetScan, and StarBase databases; mRNAs recognized by at least three databases were considered as candidate targets (Fig. S1d). After taking the intersection with 5100 DEmRNAs, only 153 DEmRNAs were identified (Fig. S1e). Finally, a total of 4 DElncRNAs, 15 DEmiRNAs, and 153 DEmRNAs were incorporated into the ccRCC-associated ceRNA regulatory network by applying Cytoscape software (Fig. S1f). To better understand the underlying function of the ceRNA network, Gene Ontology (GO) enrichment analysis and Kyoto Encyclopedia of Genes and Genomes (KEGG) pathway analysis were performed. Various cancer-related pathways were identified, such as DNA-binding transcription activator activity, approximal promoter sequence-specific DNA binding, PI3K–Akt signaling pathway, and microRNAs in cancer (Fig. S2), indicating the significant roles of the ceRNA network in the progression of cancers.

### Confirmation of the differential expression and potential prognostic value of lncRNA RCAT1 in ccRCC

**Fig. 3:**
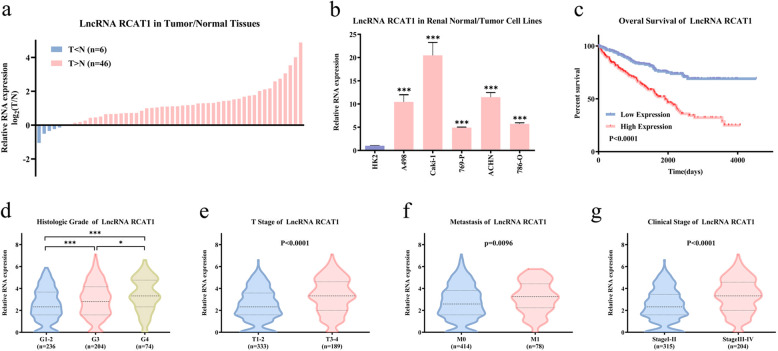
High lncRNA RCAT1 expression predicts worse prognosis of patients with ccRCC. **a** The differential expression of lncRNA RCAT1 in ccRCC tissues and normal tissues. **b** The differential expression of lncRNA RCAT1 in ccRCC cells and normal cells. **c** The Kaplan–Meier analysis was used to evaluate the relationship between lncRNA RCAT1 expression and overall survival time of ccRCC patients. **d–g** The differential expression of lncRNA RCAT1 in indicated ccRCC tissues according to TCGA database (^*^P < 0.05, ^***^P < 0.001).

Based on the above results, two lncRNAs (ENSG00000270661 and ENSG00000245694) in the ceRNA regulatory network caught our attention, which were negatively associated with the prognosis of ccRCC patients. Previous studies revealed significant role of ENSG00000245694 in various cancers [11–13], such as glioma, colorectal carcinomas, and pancreatic cancer. However, there is no report about ENSG00000270661 to date. Therefore, we selected the unannotated and poor prognosis-associated lncRNA RCAT1 (ENSG00000270661) for further investigation. lncRNA RCAT1 is located on 6q21 in humans and is composed of one exon with a full length of 1931 nt (Fig. S3a). The sequence and secondary structure of lncRNA RCAT1 are shown in Fig. S3b, c. By using several well-known methods, such as PRIDE database [14], Lee translation initiation sites [15], PhyloCSF [16], Bazzini small ORFs [17], and coding potential assessment tool [18], we found that lncRNA RCAT1 has no protein-coding potential (Fig. S3d). Consistently, there is no valid Kozak consensus sequence in lncRNA RCAT1 [19], which is essential for the initiation of translation. We further constructed a plasmid according to previous study [20], with potential Kozak sequence, a translation start codon, and a Flag tag at the 5′ terminus of the lncRNA RCAT1 and a translation stop codon at the 3′ terminus of lncRNA RCAT. However, no corresponding protein could be detected using western blot (Fig. S3e). These results collectively suggested that lncRNA RCAT1 has no protein-coding potential. We first determined the expression pattern of lncRNA RCAT1 in ccRCC. The qRT-PCR results showed that lncRNA RCAT1 expression was remarkably increased in ccRCC tissues compared to normal tissues (Fig. 3a). Moreover, compared to normal renal cells (HK2), lncRNA RCAT1 expression was significantly increased in RCC cells (A498, Caki-1, 769-p, ACHN, 786-O) (Fig. 3b). Using the median lncRNA RCAT1 expression in patients with ccRCC, patients from TCGA database were divided into high- and low-expression groups. The Kaplan–Meier survival curve showed that high lncRNA RCAT1 expression was significantly associated with poor prognosis of ccRCC patients (Fig. 3c). Moreover, the expression of lncRNA RCAT1 was upregulated in ccRCC tissues with advanced grades, larger tumor size, distant metastasis, or late clinical stages (Fig. 3d–g), indicating that lncRNA RCAT1 may function as a tumor promoter in ccRCC.

### lncRNA RCAT1 facilitates the proliferation, migration, and invasion of renal cancer cells

**Fig. 4:**
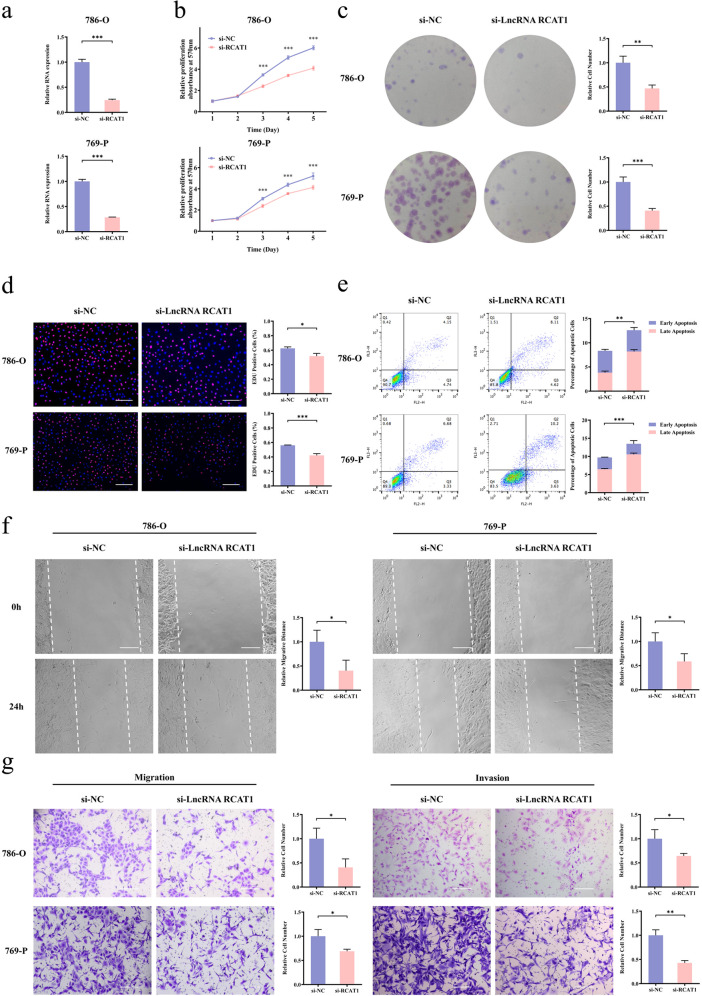
lncRNA RCAT1 knockdown suppresses malignant phenotypes in RCC cells. **a** The lncRNA RCAT1 mRNA levels in 786-O and 769-P cells transfected with lncRNA RCAT1 or negative control siRNAs. **b** The cell proliferation of 786-O and 769-P cells in response to lncRNA RCAT1 knockdown was measured using MTT assay. **c** Colony-formation assays performed with the 786-O and 769-P cells transfected with lncRNA RCAT1 or negative control siRNAs. **d** EdU assay was used to evaluate the effect of lncRNA RCAT1 knockdown on cell proliferation. Scale bar, 200 μm. **e** Apoptosis was assayed by flow cytometry in 786-O and 769-P cells after lncRNA RCAT1 knockdown. **f** The wound-healing assay was performed to examine the migration abilities after lncRNA RCAT1 knockdown. Scale bar, 200 μm. **g** The effects of lncRNA RCAT1 knockdown on migration and invasive abilities of 786-O and 769-P cells were evaluated by the transwell assays. Scale bar, 200 μm (^*^P < 0.05, ^**^P < 0.01, ^***^P < 0.001).

We further explore the biological function of lncRNA RCAT1 in RCC, small interference RNAs (siRNAs) against lncRNA RCAT1 was transfected into 786-O and 769-P cells, and the knockdown efficiency was verified by qRT-PCR assay (Fig. 4a). lncRNA RCAT1 knockdown significantly inhibited the proliferation of 786-O and 769-P cells, as determined by MTT assay, colony-formation assay, and EdU assay (Fig. 4b–d). Moreover, lncRNA RCAT1 knockdown led to an increased apoptotic rate (Fig. 4e). The wound-healing assay showed that lncRNA RCAT1 knockdown significantly inhibited cell migration (Fig. 4f). Similarly, transwell assays revealed that lncRNA RCAT1 knockdown inhibited cell migration and invasion (Fig. 4g). Consistently, ectopic expression of lncRNA RCAT1 promoted cell growth, migration and invasion, and inhibited cell apoptosis (Fig. S4). We further investigated the effect of lncRNA RCAT1 on the expression of related genes. Our results indicated that lncRNA RCAT1 knockdown led to decreased expression of Fibronectin, N-cadherin, and Vimentin, but increased expression of p53, BAX, Rb, p21, and E-cadherin (Fig. S5a). Moreover, lncRNA RCAT1 overexpression led to the opposite results (Fig. S5b). These results indicated that lncRNA RCAT1 may promote the progression of renal cancer cells in vitro.

### Identification of miR‑214‑5p as a target of lncRNA RCAT1

**Fig. 5:**
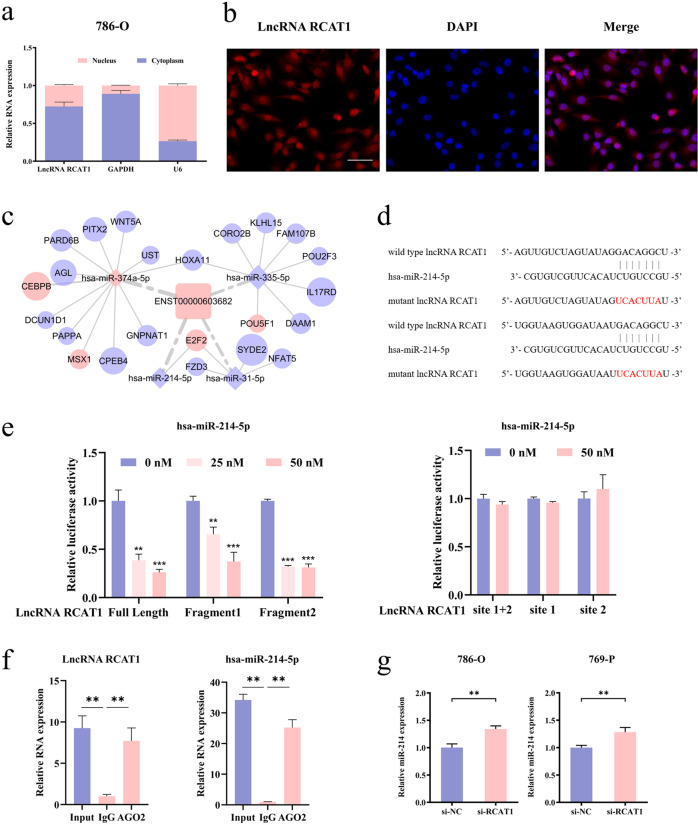
MiR-214-5p was one target of lncRNA RCAT1. **a** The subcellular location of lncRNA RCAT1 in 786-O cells. U6 and GAPDH were used as internal controls. **b** FISH assay was used to detect the subcellular location of lncRNA RCAT1 in 786-O cells. Scale bar, 2000 μm. **c** The lncRNA RCAT1-centric ceRNA network. **d** Predicted binding sites of miR-214-5p in lncRNA RCAT1 sequence. **e** Dual-luciferase assay was performed to confirm the interaction between miR-214-5p and lncRNA RCAT1. **f** The expression levels of lncRNA RCAT1 and miR-214-5p were detected in the substrate of RIP assay by qRT-PCR. **g** lncRNA RCAT1 knockdown led to increased expression of miR-214-5p (^*^P < 0.05, ^**^P < 0.01, ^***^P < 0.001).

Given that the subcellular localization of lncRNAs plays a vital role in predicting their molecular function, we firstly evaluated the subcellular localization of lncRNA RCAT1. The nuclear/cytosol fractionation assay and fluorescence in situ hybridization (FISH) assay indicated the cytoplasmic location of lncRNA RCAT1 in RCC cells (Fig. 5a, b), indicating that lncRNA RCAT1 might serve as a ceRNA for specific miRNAs. Based on the previously constructed ceRNA regulatory network, we identified that two miRNAs (miR-214-5p and miR-31-5p) might interact with lncRNA RCAT1 to regulate the expression of E2F2 (Fig. 5c). In search of direct target miRNA of lncRNA RCAT1, dual-luciferase assay was performed. The results showed that only miR-214-5p mimics significantly decreased the luciferase activities of the wild-type lncRNA RCAT1 reporter vector containing the putative miR-214-5p recognition site, but not that of the lncRNA RCAT1 reporter vector containing the mutated miR-214-5p sponging sites, indicating that miR-214-5p was probably the downstream target of lncRNA RCAT1 (Figs. 5d, e and S6a, b). Moreover, RNA immunoprecipitation (RIP) assay using antibodies against AGO2 demonstrated that lncRNA RCAT1 and miR-214-5p was preferentially enriched in AGO2-containing miRNA ribonucleoprotein complexes (miRNPs) relative to control IgG immunoprecipitates (Fig. 5f). Then, we performed a pull-down assay with a biotinylated lncRNA RCAT1 probe and the miR-214-5p with enhanced fold-change for lncRNA RCAT1 capture was observed (Fig. S6c), further confirming the interaction between lncRNA RCAT1 and miR-214-5p. Furthermore, knockdown or overexpression of lncRNA RCAT1 resulted in up- or downregulation of miR-214-5p in renal cancer cells, respectively (Figs. 5g and S6d), indicating the negative regulatory effect of lncRNA RCAT1 on the expression of miR-214-5p. These results revealed that lncRNA RCAT1 might function as a ceRNA for miR-214-5p in renal cancer cells.

### lncRNA RCAT1 exerts biological functions in renal cancer cells through regulating miR-214-5p

**Fig. 6:**
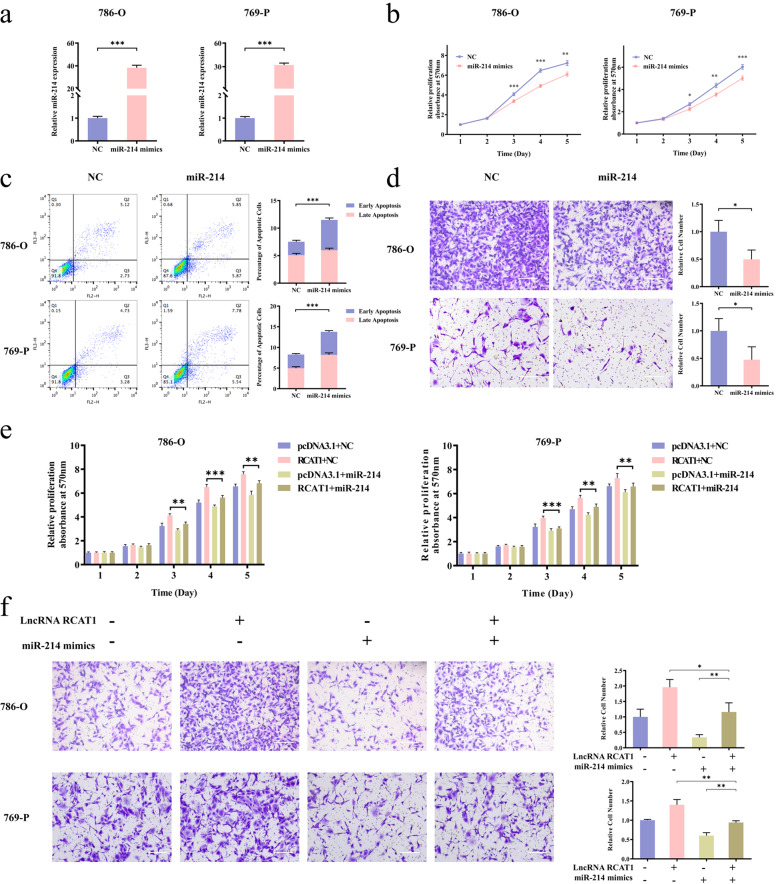
miR-214-5p negatively regulates the function of lncRNA RCAT1. **a** The efficiency of miR-214-5p mimics was detected by qRT-PCR. **b** Overexpression of miR-214-5p led to decreased cell proliferation. **c** Overexpression of miR-214-5p led to increased cell apoptosis. **d** Overexpression of miR-214-5p led to decreased cell migration. Scale bar, 200 μm. **e** Overexpression of miR-214-5p attenuated the effect on promoting proliferation of lncRNA RCAT1 by MTT assay. **f** Overexpression of miR-214-5p effectively reverses lncRNA RCAT1-induced promotion of cell migration using transwell assay. Scale bar, 200 μm (^*^P < 0.05, ^**^P < 0.01, ^***^P < 0.001).

We further evaluated the role of miR-214-5p in RCC cells. RCC cells were transfected with miR-214-5p mimics, the efficiency was detected by qRT-PCR analysis (Fig. 6a). The results showed that the RCC cell proliferation was reduced and cell apoptosis was increased by miR-214-5p overexpression (Fig. 6b, c). The transwell assay indicated that miR-214-5p overexpression led to decreased cell migration (Fig. 6d). Moreover, overexpression of miR-214-5p could restore the promotive effect of lncRNA RCAT1 on proliferation and migration of 786-O and 769-P cells (Fig. 6e, f). Above all, these data suggested that miR-214-5p served as a tumor suppressor and could partly reverse the oncogenic effect of lncRNA RCAT1 in RCC cells.

### lncRNA RCAT1 modulated E2F2 expression through sponging miR-214-5p

**Fig. 7:**
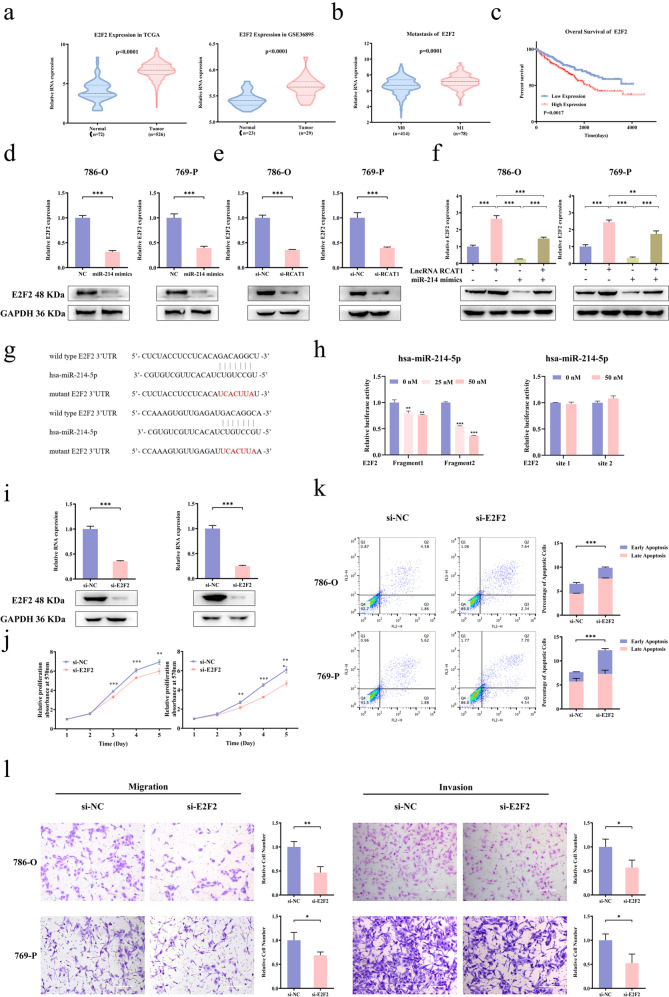
lncRNA RCAT1 promoted RCC cell progression through protecting E2F2 from miR-214-5p-induced degradation. **a** The differential expression of E2F2 in ccRCC tissues and normal tissues based on TCGA and GEO databases. **b** The differential expression of E2F2 in ccRCC tissues with or without metastasis. **c** The Kaplan–Meier analysis was used to evaluate the relationship between E2F2 expression and overall survival time of ccRCC patients. **d**, **e** Overexpression of miR-214-5p (**d**) or lncRNA RCAT1 (**e**) knockdown led to decreased mRNA and protein levels of E2F2 in RCC cells. **f** Overexpression of miR-214-5p effectively reverses lncRNA RCAT1-induced increased mRNA and protein levels of E2F2. **g** The schematic illustration showing the predicted binding sites of miR-214-5p in 3′UTR of E2F2. **h** Luciferase assay was used to show the regulatory relationship between miR-214-5p and E2F2. **i** The efficiency of E2F2 knockdown was detected by qRT-PCR and western blot. **j** Cell proliferation was evaluated after E2F2 knockdown using MTT assay. **k** Cell apoptosis was evaluated after E2F2 knockdown using flow cytometry assay. **l** Cells migration and invasion abilities were detected after E2F2 knockdown by transwell assays. Scale bar, 200 μm (^**^P < 0.01, ^***^P < 0.001).

Based on TCGA and GEO databases, the expression of E2F2 was significantly higher in renal cancer tissues compared to normal tissues (Fig. 7a). The E2F2 expression was upregulated in renal cancer tissues with metastasis compared to those without metastasis (Fig. 7b). Moreover, high expression of E2F2 was associated with poor OS of RCC patients (Fig. 7c). Compared with control group, the mRNA and protein levels of E2F2 were downregulated after miR-214-5p overexpression or lncRNA RCAT1 knockdown (Fig. 7d, e). In addition, the increased expression of E2F2 induced by lncRNA RCAT1 overexpression were diminished by miR-214-5p mimics (Fig. 7f). Luciferase reporter vectors containing wild-type or mutant miR-214-5p binding sites on the 3′UTR of E2F2 were constructed (Fig. 7g). The luciferase activity of wild-type, but not mutant E2F2 reporter, was significantly reduced by miR-214-5p mimics (Fig. 7h), indicating that miR-214-5p could bind to the 3′UTR of E2F2 mRNA. Significantly, E2F2 knockdown led to markedly decreased cell proliferation and increased cell apoptosis (Fig. 7i, k). Moreover, transwell assay revealed that E2F2 knockdown led to decreased cell migration and invasion (Fig. 7l). These findings demonstrated that lncRNA RCAT1 promoted RCC cell progression through protecting E2F2 from miR-214-5p-mediated degradation.

### lncRNA RCAT1 promotes tumor growth and metastasis in vivo

**Fig. 8:**
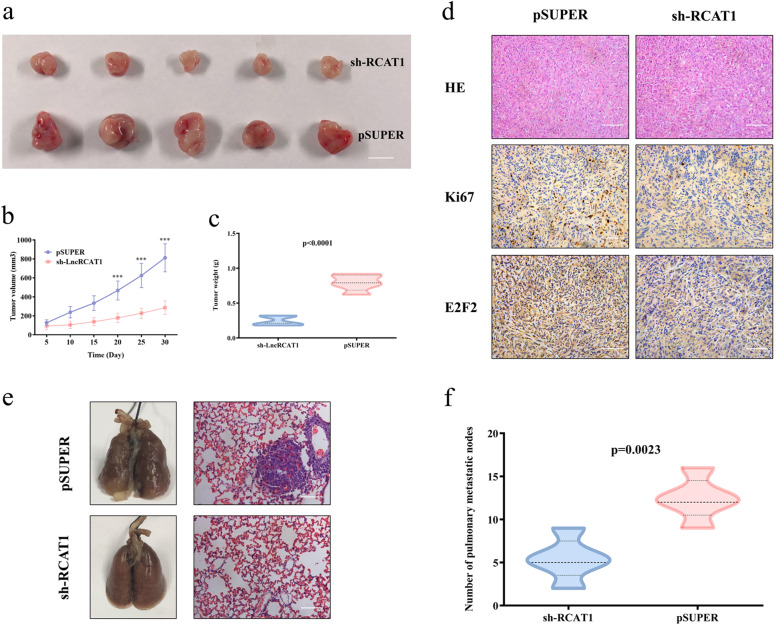
lncRNA RCAT1 knockdown suppresses tumor proliferation and metastasis in vivo. **a–c** The photos (**a**), growth curve (**b**), and weight (**c**) of the xenograft tumors. Scale bar, 1 cm. **d** H&E staining of the xenograft tumors. IHC results of Ki67 level and E2F2 level in xenograft tumor tissues. Scale bar, 100 μm. **e** Representative images of lung metastatic nodules and H&E staining of lungs isolated from nude mice (*n* = 5 for each group). Scale bar, 100 μm. **f** The numbers of metastatic nodules in the lungs of nude mice were calculated and compared. Scare bar = 50 μm (^***^P < 0.001).

We further evaluated the effect of lncRNA RCAT1 on tumor growth and metastasis in vivo. The xenograft experiment results revealed that lncRNA RCAT1 knockdown led to reduced proliferation of tumors and downregulated expression of cell proliferation marker Ki67 (Fig. 8a–d). The expression of E2F2 was also decreased in xenograft tumor tissues of lncRNA RCAT1 knockdown group as determined by immunohistochemistry (IHC) and qRT-PCR assays (Figs. 8d and S6a). Moreover, qRT-PCR assays revealed that lncRNA RCAT1 expression was decreased and miR-214-5p expression was increased in xenograft tumor tissues of lncRNA RCAT1 knockdown group (Fig. S6b, c). These results further indicated that lncRNA RCAT1 promotes cell proliferation by regulating miR-214-5p/E2F2 axis. To determine the effect of lncRNA RCAT1 on RCC metastasis in vivo, we established a lung metastasis mouse model. The number and diameter of pulmonary metastasis lesions were smaller and fewer in the lncRNA RCAT1 knockdown group (Fig. 8e, f). These results suggest that lncRNA RCAT1 could promote RCC tumor growth and metastasis in vivo.

## Discussion

As the most prevalent type of RCC, ccRCC has an increasing incidence and higher mortality rate. Given that the symptoms of ccRCC are insidious in the early stages and the sensitivity to chemotherapy and radiation therapy of ccRCC is extremely low, the survival rate of ccRCC patients is still unsatisfied [21]. Therefore, a comprehensive understanding of the molecular mechanisms and identification of novel therapeutic targets and prognostic biomarkers for ccRCC is urgently needed.

Aberrantly expressed lncRNAs have been found in various cancers and the expression of lncRNAs was associated with the outcomes of cancers [22, 23]. For example, a six‐lncRNA signature was constructed in glioblastoma to predict prognosis [24]. In addition, a risk evaluating model based on three lncRNAs was developed to predict the prognosis of patients with esophageal squamous cell cancer [25]. However, a comprehensive lncRNA-based prognostic prediction model in ccRCC has not been clearly elucidated. In this study, we screen the expression of lncRNAs in ccRCC patients from GEO and TCGA databases. Using Cox regression model and Lasso analyses, five prognosis-related lncRNAs were identified to construct a novel lncRNA-based prognostic prediction model, which classified ccRCC patients into high-risk or low-risk groups. The predictive accuracy was further validated by Kaplan–Meier analysis and ROC curve analysis, highlighting that lncRNAs might be prospective markers for prognosis prediction of ccRCC patients.

Various studies reveal that lncRNAs exert their effects on cancer cells through various mechanisms, especially ceRNA networks. Based on the results of bioinformatics analysis and database prediction, we established a lncRNA–miRNA–mRNA regulatory network. The GO and KEGG pathway analysis revealed that the function of the ceRNA network might be related with the PI3K–Akt signaling pathway, which is associated with various cancer-related biological processes [26], such as cell proliferation, apoptosis, and motility. In ccRCC, the PI3K–Akt signaling pathway is constitutively activated and shows critical role in cancer progression through regulating various targets, such as bromodomain-containing protein 4 (BRD4) [27] and VHL–HIF pathway [28]. However, the prognostic role of PI3K/AKT signaling pathway was controversial [29–31], which might be related with the specific regulatory pathways, heterogeneity of RCC patients, and limited sample size. More investigations are needed to further elucidate the prognostic roles of PI3K/AKT signaling pathway.

Based on the prognostic model and ceRNA network, two lncRNAs were identified, which were negatively associated with the prognosis of ccRCC patients. ENSG00000245694 (also named CRNDE) has been fully investigated in various cancers, however, there was no report for ENSG00000270661 (lncRNA RCAT1). Therefore, lncRNA RCAT1 was selected for further investigation. However, the biological functions and regulatory mechanisms of other lncRNAs in ccRCC are needed to be fully elucidated in the future. Our results revealed that lncRNA RCAT1 was significantly upregulated in ccRCC tissues and associated with poor prognosis of ccRCC patients. Moreover, in vitro and in vivo experiments demonstrated that lncRNA RCAT1 could promote ccRCC cell proliferation, migration, and invasion, revealing an oncogenic role of lncRNA RCAT1 in ccRCC. Previous studies revealed the significant association between the subcellular location of lncRNAs and their potential regulatory mechanisms. The results of subcellular fractionation indicated the cytoplasmic distribution of lncRNA RCAT1, implying the potential for serving as a ceRNA. Further luciferase reporter assay and RIP assay indicated that lncRNA RCAT1 could sponge miR-214-5p, and lncRNA RCAT1 knockdown led to an increase in the expression of miR-214-5p, indicating a negative correlation between them. The aberrant expression and tumor-suppressive role of miR-214-5p had been reported in various cancers, such as non-small lung cancer [32], esophageal cancer [33], and cervical cancer [34]. However, the role of miR-214-5p in osteosarcoma is inconsistent [35–38], which might be attributed to specific biological signaling. In addition, there is no related research about the role of miR-214-5p in RCC. In our study, we demonstrated that miR-214-5p overexpression could inhibit proliferation and metastasis of RCC cells, indicating a tumor-suppressive role of miR-214-5p. Moreover, miR-214-5p could partly abolish the lncRNA RCAT1-mediated malignant biological effects. These data strongly demonstrated that lncRNA RCAT1 served as a sponge for miR-214-5p. Various studies also revealed the significant role of protein–lncRNA interaction in the regulation of tumorigenesis and cancer progression. lncRNA AATBC could promote breast cancer migration and invasion through binding with YBX1 to activate the YAP1/Hippo signaling pathway [39]. lncRNA TP53TG1 suppressed the progression of hepatocellular carcinoma through interacting with PRDX4 to promote its ubiquitin-mediated degradation, leading to inactivation of the WNT/β-catenin pathway [40]. Moreover, one lncRNA might participate in the tumor progression through various mechanisms. For example, the well-characterized lncRNA HOTAIR could promote proliferation and metastasis of breast cancer through either regulating miR-20a-5p/HMGA2 pathway [41] or enhancing the ER expression and ER occupancy on its downstream target genes [42]. However, the protein-binding potential of lncRNA RCAT1 and the detailed mechanisms need to be further elucidated. In addition, we also detected the distribution of lncRNA RCAT1 in the nucleus. Previous studies revealed that lncRNAs located in the nucleus exert critical roles in regulating gene expression through various nuclear events [43], such as transcriptional regulation, RNA processing, and chromatin remodeling. lncRNA HIFAL could introduce the PKM2/PHD3 complex into nucleus through binging with hnRNPF to promote the transactivation of HIF-1α [44]. lncRNA PRADX promoted the H3K27me3 in the UBXN1 promoter via binding with EZH2 to recruit PRC2/DDX5 complex [45]. Therefore, lncRNA RCAT1 might have putative role in transcriptional processing with protein-binding potential to promote cancer progression. Recently, several studies revealed the significant effectiveness of RNA-based therapeutic approaches [46, 47], such as delivery of antisense oligonucleotides and RNA interference (RNAi) therapy, providing promising approaches for cancer control. Moreover, the advancement in synthetic delivery carriers or chemical modifications brought more promising results. Previous study revealed a nanoparticle-mediated RNAi targeting oncogenic lncRNA DANCT, with efficient cellular uptake, sustained target silencing, and no overt toxic side-effects, which could suppress cancer cell proliferation and progression in vitro and in vivo [48]. Recently, Battistelli et al. demonstrated the effectiveness of a novel negative-based RNA strategy. They constructed an HOTAIR deletion mutant form [49], named HOTAIR-sbid, which retained the combining ability with Snail but depleted the EZH2-binding domain. The HOTAIR-sbid could inhibit malignant phenotypes of cancer cells through competitively binding with Snail and enhancing the EZH2-mediated repression on Snail epithelial target genes. Although we did not identify strong association between lncRNA RCAT1 and EZH2 or Snail based on catRAPID database [50], other binding proteins might serve as the targets of lncRNA RCAT1 considering its nuclear distribution and protein-binding potential. Our results identified lncRNA RCAT1 as a vital oncogenic gene in renal cancer, it has the potential to be a promising candidate targeting multiple signaling pathways to overcome the limitations of single-target therapies. However, more studies were needed to further elucidate the underlying mechanism and therapeutic potential of lncRNA RCAT1 in the future.

Previous studies revealed that the E2F family of transcription factor 2 (E2F2) plays a crucial role in the development and progression of various cancers, such as ovarian cancer [51], lung cancer [52], and hepatocellular carcinoma [53], promoting cell-cycle progression, stemness, metastasis, and chemoresistance [54]. The upregulated E2F2 was also correlated with higher grade of tumors and unfavorable prognosis [51]. Some evidence showed that E2F2 was increased in ccRCC tissues [55], however, the roles of E2F2 in ccRCC have not been well characterized. Our current study demonstrated that E2F2 was upregulated in ccRCC tissues and E2F2 knockdown could inhibit proliferation, migration, and invasion of ccRCC cells. Moreover, our comprehensive bioinformatics analysis combined luciferase reporter assay and RIP experiments verified miR-214-5p as a sponge target of both lncRNA RCAT1 and E2F2, and the expression of E2F2 was modulated by lncRNA RCAT1/miR-214-5p axis.

In summary, the present work is the first systematic study about the role of lncRNA RCAT1 as a ceRNA, and the interactions among lncRNA RCAT1, miR-214-5p, and E2F2 are responsible for the lncRNA RCAT1-mediated ccRCC progression. Our research contributes to a more comprehensive understanding of ccRCC progression and provides a basis for development of effective novel therapeutic targets and reliable prognostic predictors for ccRCC.

## Materials and methods

### Data collection

The high-throughput sequencing data used in the current study were acquired from GEO (http://www.ncbi.nlm.nih.gov/gds/) and TCGA (https://portal.gdc.cancer.gov/). The expression data of lncRNA were obtained from GSE96574 (5 pairs of ccRCC tissues and normal tissues) and TCGA (526 ccRCC tissues and 72 normal tissues). The miRNA (512 ccRCC tissues and 71 normal tissues) and mRNA (526 ccRCC tissues and 72 normal tissues) expression data were derived from TCGA. Clinical data of ccRCC patients were also acquired from the TCGA database.

### Differential expression analysis of lncRNAs, miRNAs, and mRNAs

The analysis of differentially expressed lncRNAs (DElncRNAs), miRNAs (DEmiRNAs), and mRNAs (DEmRNAs) between tumor samples and normal samples was performed using the limma package of R software. The criteria for selection of DElncRNAs, DEmiRNAs, and DEmRNAs were *P*-value <0.05 and | log_2_ fold change (FC) | > 1. The heatmap and volcano plot were drawn using the heatmap package and ggplot2 package, respectively.

### Construction and validation of the lncRNA‑related prognostic model

A total of 522 ccRCC patients were firstly subjected to univariate Cox regression analysis to evaluate the prognostic value of the DElncRNAs for OS. The lncRNAs were regarded as significant at *P* < 0.05. Then, the least absolute shrinkage and selection operator (LASSO) model was used to further select crucial prognostic lncRNAs. We subsampled the dataset 1000 times and chose the lncRNAs that were repeated >10 times. Next, the selected lncRNAs were subjected to multivariate Cox regression analysis in which lncRNAs were regarded as significant at *P* < 0.05. The lncRNA‐related prognostic model was constructed using the regression coefficients. The formula of prognostic risk score for predicting OS was calculated as follows:

$${\mathrm{Right}}\,{\mathrm{score}} = \mathop {\sum}\limits_{i = 1}^N {{\upbeta }} \ast E$$

(*N* = the number of selected lncRNAs, *E* = expression level of lncRNAs, β = regression coefficient of lncRNAs).

The 522 ccRCC patients was classified into the low‐risk and high‐risk groups and heatmap package was used to cluster the expression profiles of lncRNAs in two groups. The ROC curve analysis and Kaplan–Meier survival analysis were performed to evaluate the sensitivity and specificity of the lncRNA‑related prognostic model in predicting ccRCC patient prognosis.

### Construction of the ceRNA network

The lncRNA–miRNA interactions were predicted using the DIANA-LncBase v2 database [56]. The miRNA–mRNA interactions were predicted using the miRDB, miRTarBase, TargetScan, and StarBase databases [57–60]. Only mRNAs recognized by at least three databases were considered as candidate targets. Cytoscape software was utilized to visualize and construct the ceRNA network [61].

### Functional enrichment analysis

In order to better understand the underlying function of DEmRNAs in the ceRNA network, the GO and KEGG analyses were carried out by utilizing ClusterProfiler package in R studio. The criterion of *P*-value was less than 0.05.

### Tissue collection

In all, 52 pairs of ccRCC and paired normal tissue samples were obtained from ccRCC patients who underwent surgery at the Shandong Cancer Hospital. Written informed consent was obtained from all patients. The experimental procedures were approved by the Institutional Ethics Committee of the Shandong Cancer Hospital. All tissue was stored at −80 °C until RNA extraction.

### Cell culture and transfection

The human RCC cell lines (ACHN, 786-O, 769-P, Caki-1, and A498) and a normal human renal cell line (HK-2) were obtained from ATCC (Manassas, USA). The cells were cultured routinely in DMEM or RPMI-1640 medium containing 10% fetal bovine serum (FBS) at 37 °C with 5% CO_2_. All of the cells were authenticated by short tandem repeat DNA fingerprinting and tested for mycoplasma.

The siRNAs targeting lncRNA RCAT1 or E2F2 and miR-214-5p mimics (Table S3) were purchased from RiboBio Company (Guangzhou, China). Full-length pf lncRNA RCAT1 was cloned into pcDNA3.1 (Invitrogen, USA) to generate pcDNA3.1-RCAT1 constructs. Lipofectamine 2000 (Invitrogen) was used for cell transfection.

### RNA extraction and qRT-PCR analysis

Total RNA from tissues and cells was extracted using Trizol reagent (Takara, Japan). The mRNA was reverse transcribed into cDNAs using the PrimeScript reverse transcriptase reagent kit (Takara, Japan) and detected by qRT-PCR using SYBR Green (Takara, Japan). The expression levels of lncRNAs and mRNAs were normalized to β-actin, while the expression levels of miR-214-5p was calculated relative to expression of U6. The data were calculated by the 2^−ΔΔCt^ method. The primers used in this study were listed in Table S4.

### Western blotting (WB)

Protein samples from cells were extracted by RIPA buffer with protease inhibitor (Beyotime, Beijing) and separated using SDS-PAGE gels. After blocking with 5% no-fat milk solution, the PVDF membranes were incubated with primary antibodies overnight at 4 °C and corresponding secondary antibodies for 2 h at room temperature. The signals were visualized using enhanced chemiluminescence (Millipore). The primary antibodies and secondary antibodies used were available in Table S5.

### Cell proliferation assay

In all, 1.5 × 10^3^ transfected cells/well were seeded into 96-well plates and incubated for indicated time. The wells received 20 μl MTT (5 mg/ml) and the plates were incubated for 4–6 h at 37 °C. Then, the medium was removed and 100 μl DMSO was added into each well. Cell proliferation was estimated by measuring absorbance at 570 nm.

### Colony-formation assay

The transfected cells were seeded into 6-well plates at a density of 1000 cells per well and were allowed to grow for at least 2 weeks until the visible colonies were formed. Then, the plates were washed with PBS, fixed with methanol, and stained with 0.2% crystal violet. The stained colonies were photographed and counted.

### EdU incorporation assay

In all, 1 × 10^4^ transfected cells/well were seeded into 96-well plates and incubated with 50 mM EdU for 2.5 h. The cells were fixed with 4% paraformaldehyde (PFA) and stained with Apollo Dye Solution and Hoechst using EdU incorporation assay kit (RiboBio, China). The images were obtained using the fluorescence microscope (Nikon, Japan).

### Flow cytometry analysis

The cell apoptosis was examined using Annexin V-FITC Apoptosis Detection Kit (BD Biosciences, USA) according to the manufacturer’s instruction. The collected cells were washed with cold PBS and resuspended in 1 X binding buffer. After incubation with Annexin V-FITC/7AAD for 30 min at room temperature in the dark, the cells were analyzed on a FACSCalibur (BD Biosciences, USA).

### Wound-healing assay

The transfected cells were seeded into 24-well plates and allowed to grow until the plates were confluent. A 10 μl pipette tip was used to create straight scratches on the cell monolayers. Then, the cells were washed with PBS and cultured in serum-free media. Images were captured at indicated time after the initial scratches.

### Transwell assay

The cell migration and invasion abilities were evaluated with transwell chambers (Corning) without coating or coated with Matrigel (BD Biosciences, USA), respectively. In all, 1 × 10^5^ cells were suspended in serum-free RPMI-1640 medium and added into the upper chambers, and 700 μl RPMI-1640 medium supplemented with 20% FBS was added to the bottom chamber. After 24 h, the cells on the lower surface were fixed with methanol, stained with 0.2% crystal violet, photographed, and counted.

### Nuclear–cytoplasmic fractionation

Separation of nuclear and cytoplasmic RNA was performed using PARIS Kit (Life Technologies, USA) according to the manufacturer’s instructions. The GAPDH was used as a cytoplasmic control and U6 was used as a nuclear control.

### Fluorescence in situ hybridization (FISH)

The Cy3-labeled lncRNA RCAT1 probes were designed and synthesized by GenePharma (Shanghai, China). The signals of the probe were detected using FISH Kit (GenePharma, China) according to the manufacturer’s instructions. All images were acquired using the fluorescence microscope (Nikon, Japan).

### Dual-luciferase reporter assay

The wild-type or mutant lncRNA RCAT1 sequence or 3′UTR of E2F2 was cloned into pmirGLO reporter vectors. The luciferase constructs were cotransfected with miR-214-5p or miR-31-5p mimics or miR-NC into HEK293T cells. After 48 h, the luciferase activities were measured using the Dual-Luciferase Reporter Assay System (Promega, USA). Relative luciferase activity was calculated by normalizing Firefly luciferase activity to Renilla luciferase activity.

### RNA immunoprecipitation (RIP)

EZ-Magna RIP RNA-Binding Protein Immunoprecipitation Kit (Millipore) was used according to the manufacturer’s instructions. Total RNAs (input controls) and corresponding species’ IgG controls were assayed simultaneously to demonstrate that the detected signals were from the RNAs that were specifically bound to AGO2 (Cell signaling, USA, Cat#2897). The presence of lncRNA RCAT1 and miR-214-5p was analyzed by qRT-PCR.

### Pull-down assay with a biotinylated lncRNA RCAT1 probe

A pull-down assay was performed as previously described [62]. Briefly, 1 × 10^7^ cells were collected, lysed, and sonicated. After coincubation of the lncRNA RCAT1 probe (RiboBio, Guangzhou, China) with C-1 magnetic beads (Life Technologies), the probe-coated beads were generated. Then, the cell lysates were incubated with the lncRNA RCAT1 probe or oligo probe at 4 °C overnight. After washing, elution, and extraction, the RNAs were used for qRT-PCR to detect the expression levels.

### Tumor xenograft model

Female BALB/c nude mice (4–6-week-old) were randomly divided into two groups, and 1 × 10^7^ stably transfected cells were subcutaneously injected into the mice (*n* = 5) to evaluate the tumorigenic effects of lncRNA RCAT1. After 30 days, the mice were sacrificed and the maximum (*L*) length, minimum (*W*) length, and weight of the tumors were measured. No blinding was performed for the animal experiments. Tumor volume was calculated as: TV = ½*LW*^2^. To evaluate the effect of lncRNA RCAT1 on metastasis, 5 × 10^5^ stably transfected cells were injected into the tail veins of nude mice (*n* = 5). After 4 weeks, the mice were killed and the lungs were collected to evaluate the number and size of pulmonary metastatic foci. Hematoxylin and eosin (H&E) staining was performed for tissue morphology evaluation. The animal experiments were approved by the Shandong University Animal Care and Use Committee.

### Immunohistochemistry (IHC)

The sections were dewaxed with xylene, rehydrated with alcohol, and heated with microwave for antigen retrieval. Then the sections were blocked with 5% BSA and incubated with anti-E2F2 antibody or anti-Ki67 antibody overnight at 4 °C. After incubating with corresponding secondary antibodies for 2 h at 37 °C, the sections were stained with diaminobenzidine and counterstained with hematoxylin. Olympus light microscope was used to take images.

### Statistical analysis

The statistical analysis was performed using SPSS 19.0 (Chicago, IL, USA). Data were presented as mean ± standard deviation (SD) from three independent experiments. Student’s *t* test was used to assess the differences between two groups. The data in statistical tests are conformed to normal distribution and the variance are similar. Comparisons among multiple groups were performed with one-way ANOVA test. The Kaplan–Meier method and log-rank test was used for survival analysis. A value of *P* < 0.05 was considered statistically significant.

## Supplementary information

Supplementary Figure.

Table S1.

Table S2.

Table S3.

Table S4.

Table S5.
