# Supplementary material for: LncRNA RCAT1 promotes tumor progression and metastasis via miR-214-5p/E2F2 axis in renal cell carcinoma
Source: Cell Death Dis. 2021 Jul 9;12(7):689. doi: 10.1038/s41419-021-03955-7 (PMC8270952; doi:10.1038/s41419-021-03955-7)
Supplement: Supplementary file 1 — Supplementary Figure. [file 41419_2021_3955_MOESM1_ESM.docx]

**Supplementary Figures and Figure Legends**

**
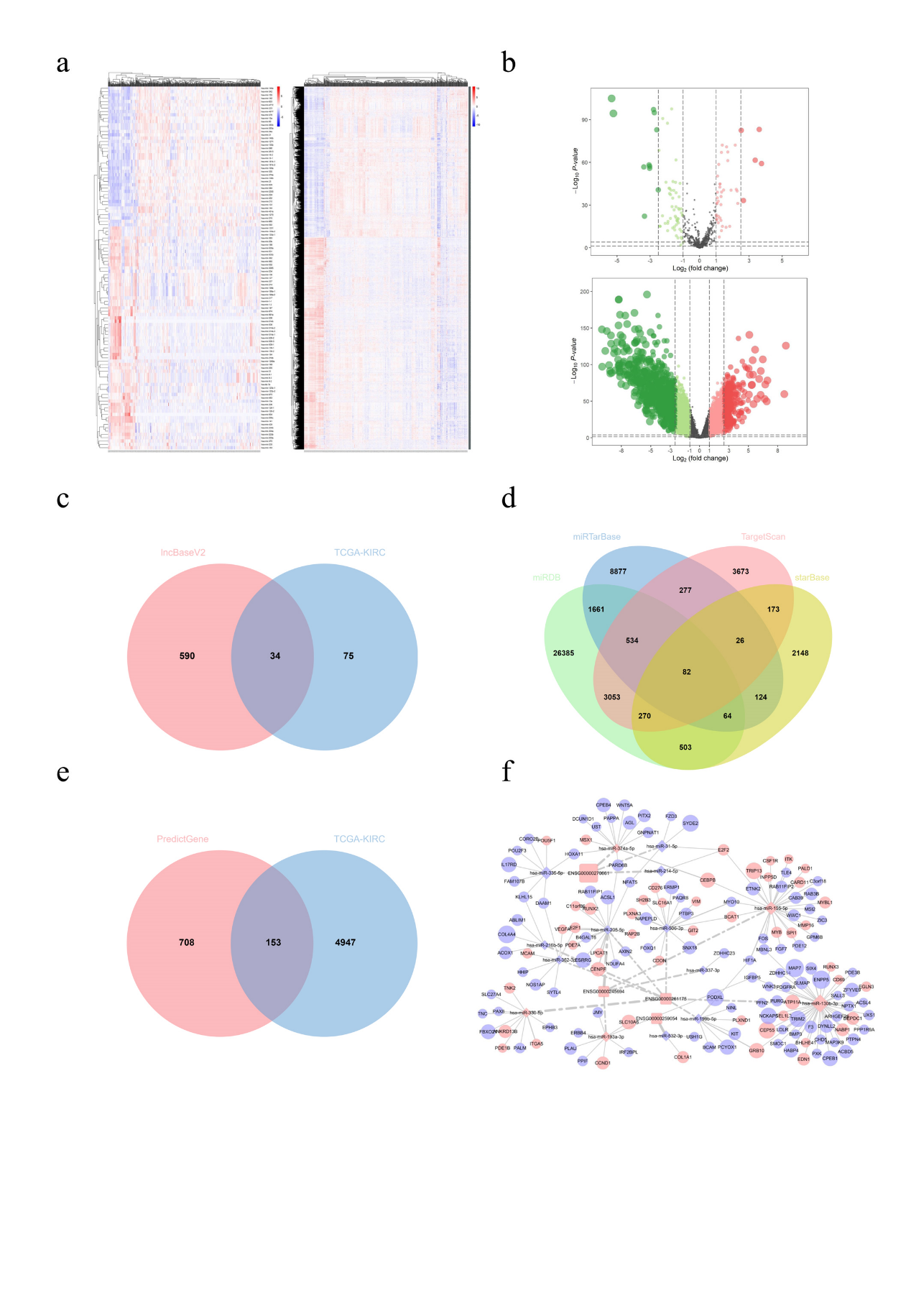
**

**Fig. S1** **Identification of differentially expressed miRNAs and mRNAs and construction of the ceRNA network. a-b** The heat maps (a) and volcano plots (b) of DEmiRNAs and DEmRNAs between ccRCC tissues and normal tissues according to TCGA database. **c** The identification of DEmiRNAs for the ceRNA network. **d** Schematic illustration showing overlapping of the target genes of miR-214-5p predicted by miRDB, miRTarBase, TargetScan, and starBase. **e** The identification of DEmRNAs for the ceRNA network. **f** The ceRNA regulatory network in ccRCC. Red rectangles represent lncRNAs. Purple diamonds and circles represent downregulated miRNAs and mRNAs, respectively. Red diamonds and circles represent upregulated miRNAs and mRNAs, respectively. Gray lines represent lncRNA-miRNA-mRNA interactions.

**
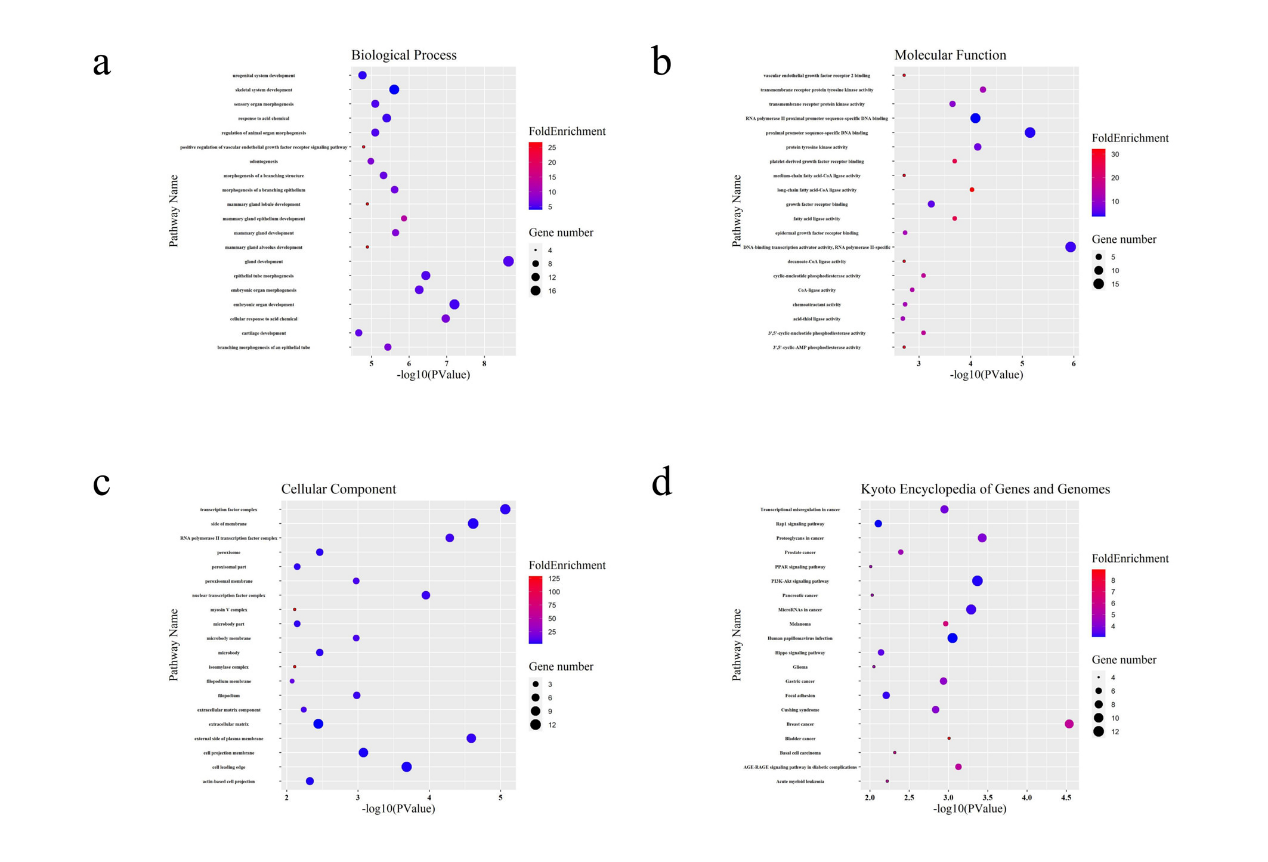
**

**Fig. S2 Functional enrichment analysis of the ceRNA network. a-d** Gene ontology (GO) analysis and KEGG analysis of DEmRNAs were used to demonstrate their potential biological functions.

**
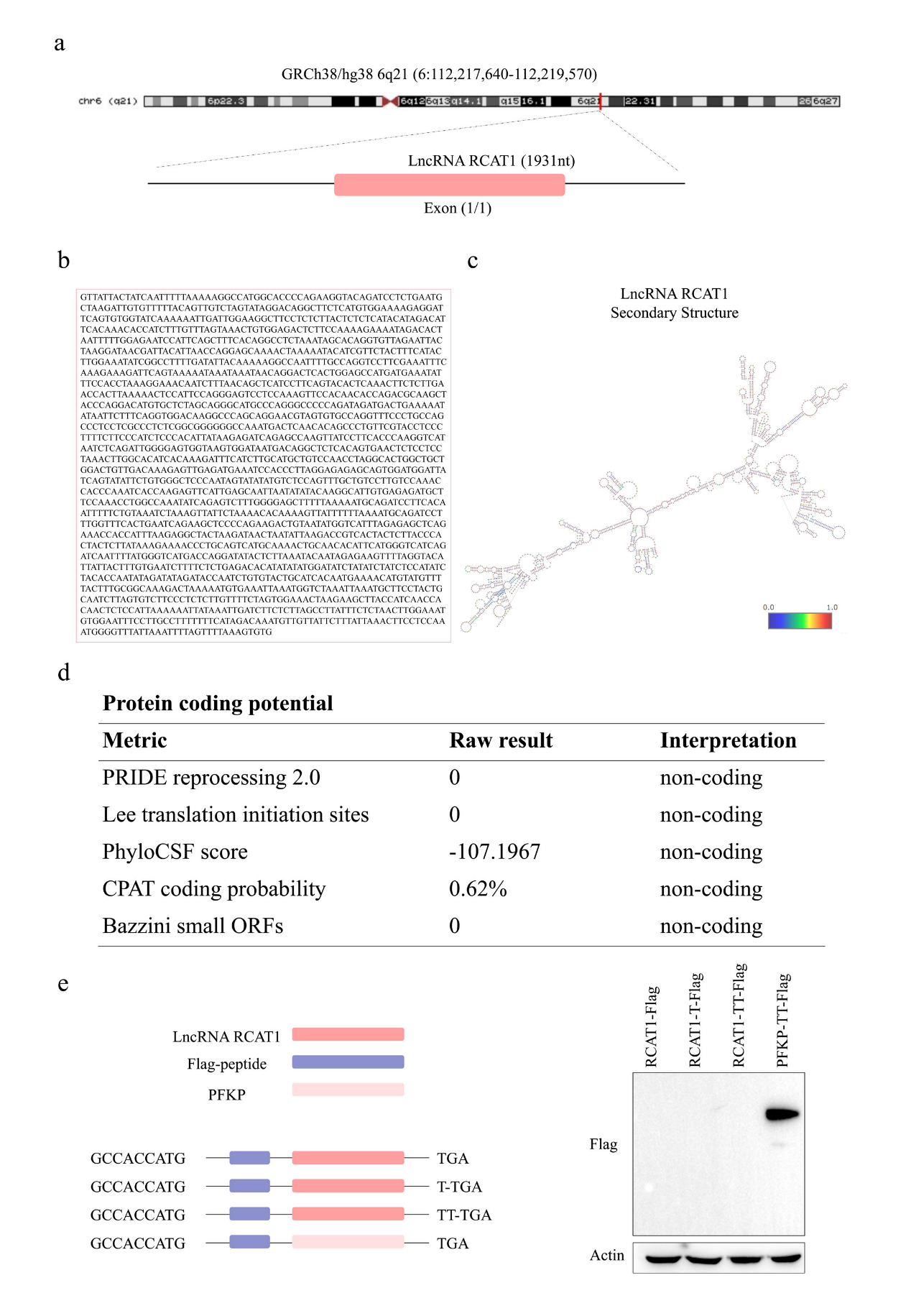
**

**Fig. S3 The basic information of lncRNA RCAT1. a** The genomic locus in humans of lncRNA RCAT1 was showed in the schematic diagram. Blue rectangles represent exons. **b** The sequence of lncRNA RCAT1. **c** The secondary structure of lncRNA RCAT1 was got from AnnoLnc (http://annolnc.cbi.pku.edu.cn/). **d** The coding potential analysis of lncRNA RCAT1 was measured by 5 kinds of metrics. **e** The schematic diagram of the constructed plasmids. PFKP with N-terminal Flag tag was used as a positive control. Anti-flag antibody was used to detect the putative proteins.

**
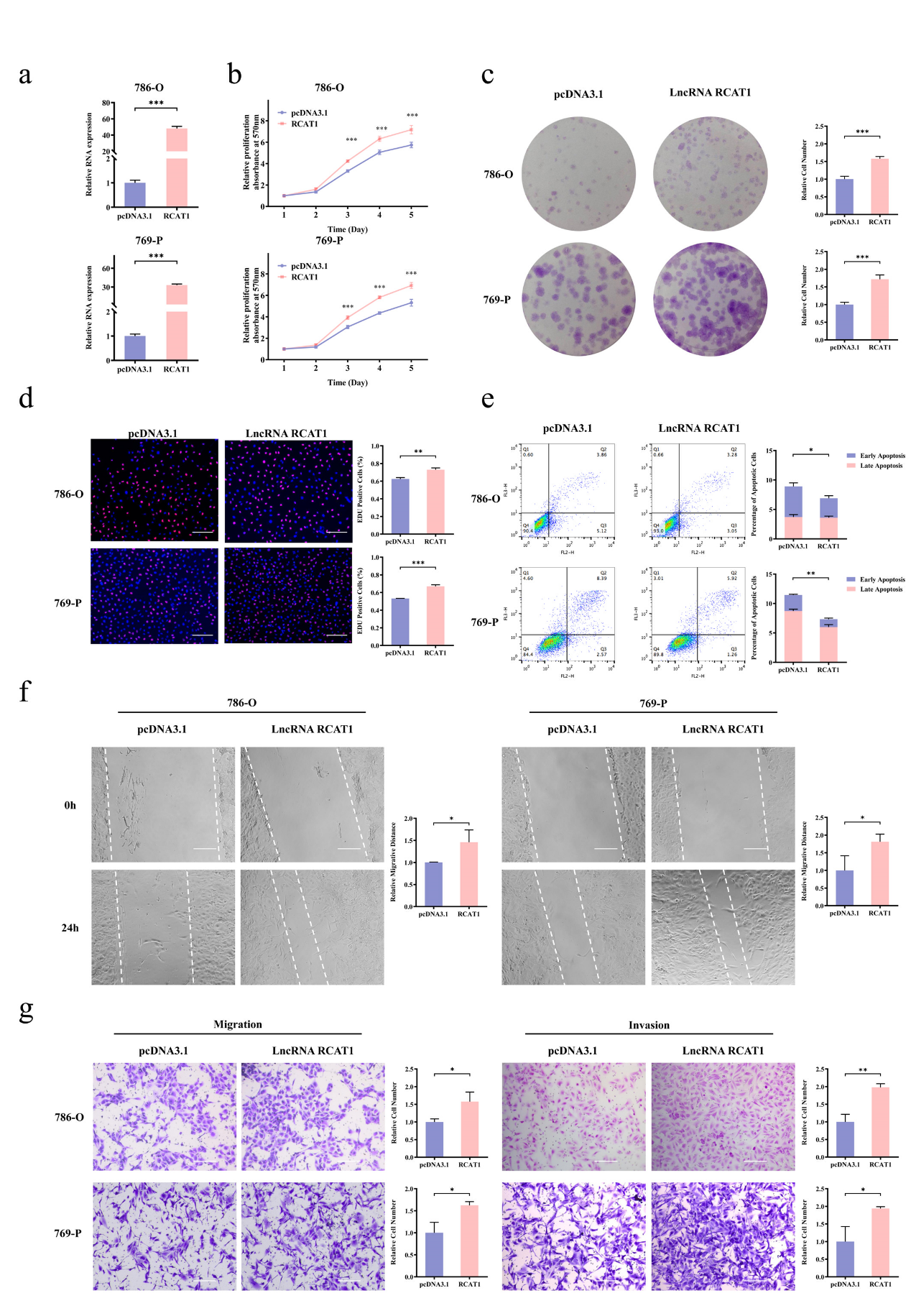
**

**Fig. S4 LncRNA RCAT1 promotes the proliferation, migration, and invasion in ccRCC cells. a** The qRT-PCR assays for the lncRNA RCAT1 levels in 786-O and 769-P cells transfected with pcDNC3.1-RCAT1 or the empty vector. **b** The cell proliferation of 786-O and 769-P in response to lncRNA RCAT1 overexpression was measured using MTT assay. **c** Colony formation assays performed with the 786-O and 769-P cells transfected with pcDNC3.1-RCAT1 or the empty vector. **d** The EdU assay was used to evaluated the effect of lncRNA RCAT1 overexpression on cell proliferation. Scale bar, 200 μm. **e** Apoptosis was assayed by flow cytometry in 786-O and 769-P cells after overexpression of lncRNA RCAT1. **f-g** Effects of lncRNA RCAT1 overexpression on migration and invasive abilities of 786-O and 769-P cells were measured by wound healing assay (f) and transwell assay (g). Scale bar, 200 μm. (*P < 0.05, **P <0.01, and ***P < 0.001)

**
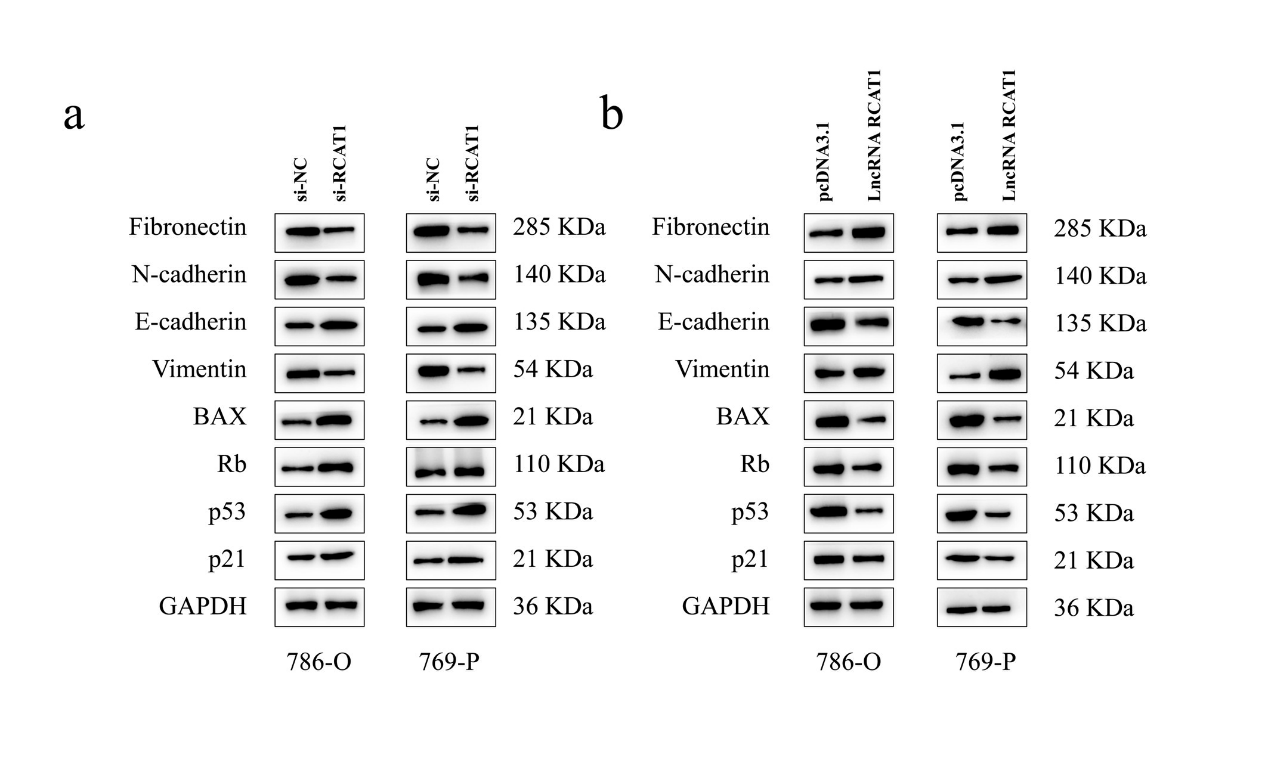
**

**Fig. S5 The effect of lncRNA RCAT1 on the expression of genes involved in cell proliferation, migration, invasion, and apoptosis. a-b** The effect of lncRNA RCAT1 knockdown (a) or overexpression (b) on the expression of Fibronectin, N-cadherin, Vimentin, E-cadherin, p53, BAX, Rb, and p21.

**
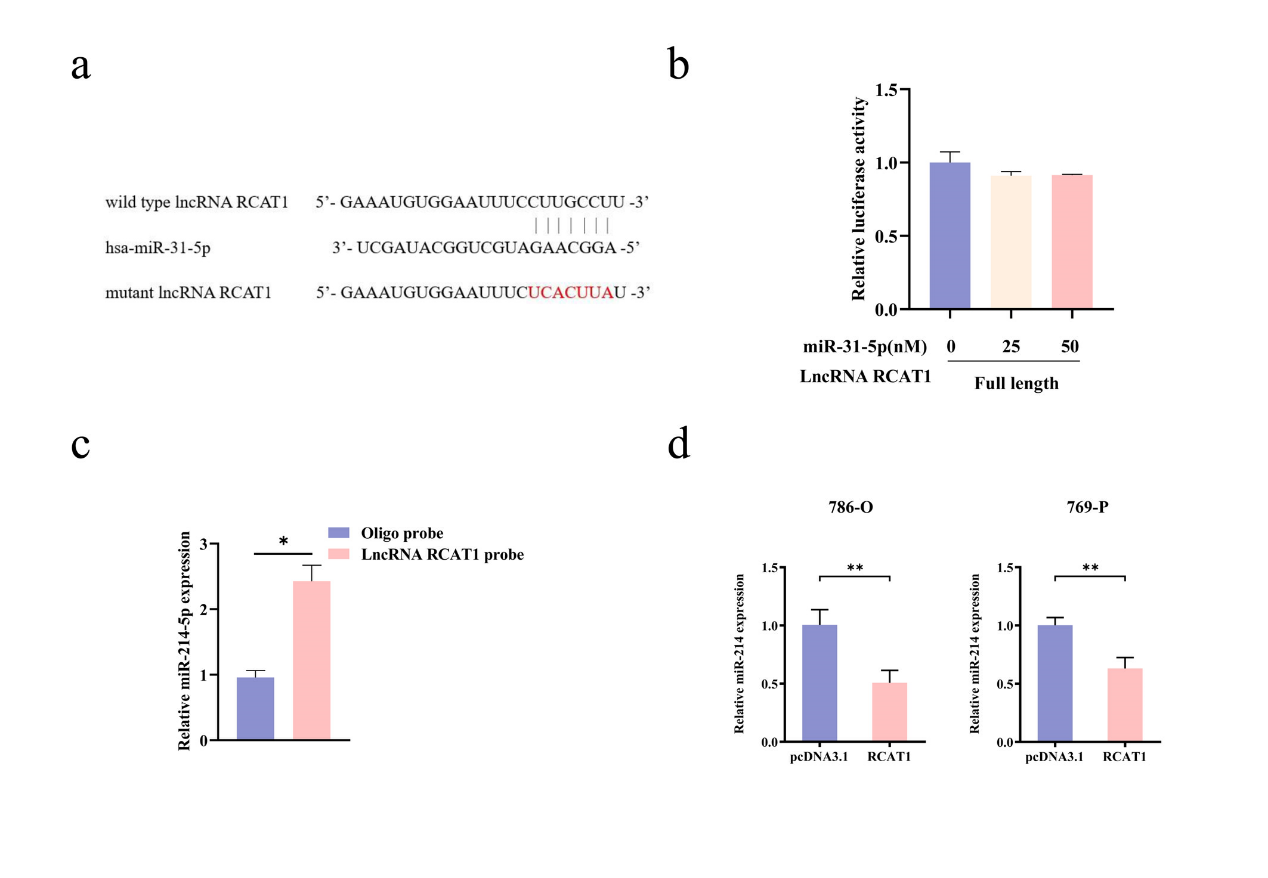
Fig. S6 LncRNA RCAT1 acts as a sponge for miR-214-5p in ccRCC cells. a** Predicted binding sites of miR-31-5p in lncRNA RCAT1 sequence. **b** Dual-luciferase assay was performed to evaluate the interaction between miR-31-5p and lncRNA RCAT1. **c** The relative levels of miR-214-5p with lncRNA RCAT1 or oligo probe were examined by qRT-PCR. **d** LncRNA RCAT1 overexpression led to decreased expression of miR-214-5p. (*P < 0.05 and **P <0.01)


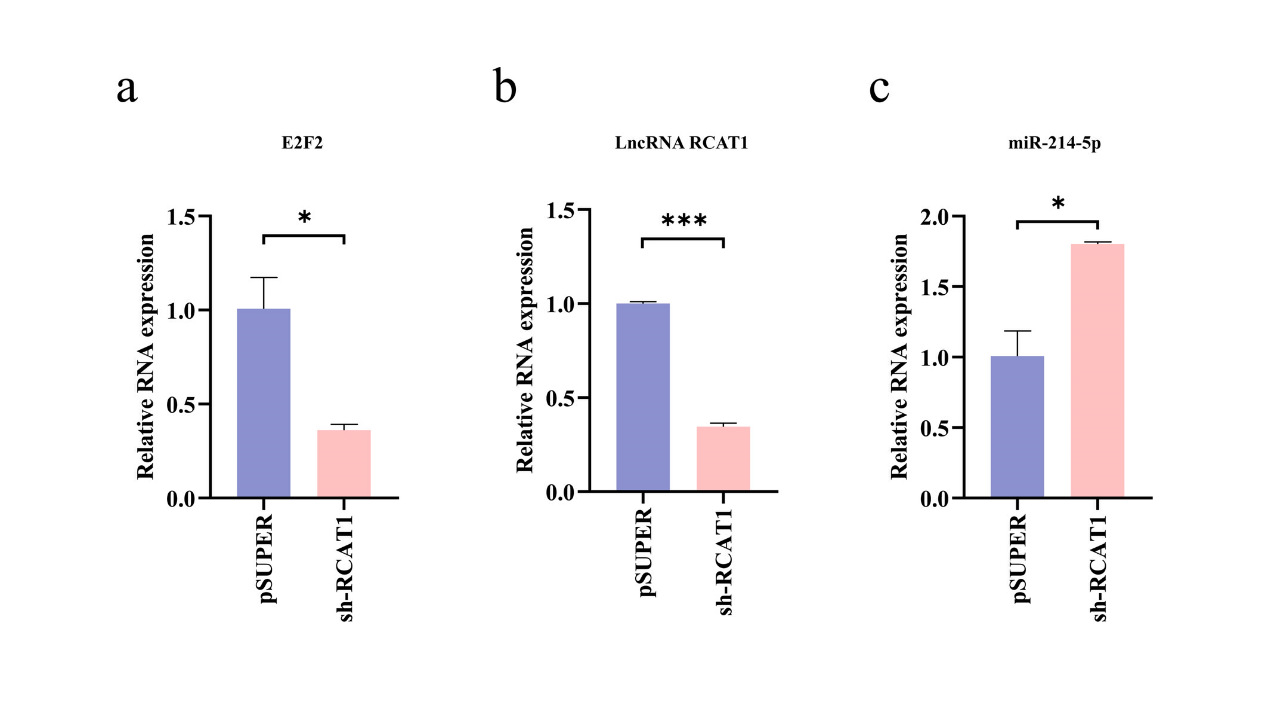


**Fig. S7 LncRNA RCAT1 regulates the expression of E2F2 and miR-214-5p in vivo. a-c** The qRT-PCR assay was used to detected the expression of E2F2, lncRNA RCAT1, and miR-214-5p in xenograft tumor tissues. (*P < 0.05 and ***P < 0.001)
