# Supplementary material for: LncRNA RCAT1 promotes tumor progression and metastasis via miR-214-5p/E2F2 axis in renal cell carcinoma
Source: Cell Death Dis. 2021 Jul 9;12(7):689. doi: 10.1038/s41419-021-03955-7 (PMC8270952; doi:10.1038/s41419-021-03955-7)
Supplement: Supplementary file 3 — Table S2. [file 41419_2021_3955_MOESM3_ESM.docx]

**Table S2. P values and regression coefficients of 5 lncRNAs.**

| **LncRNA** | **P value** | **Coefficient** |
| --- | --- | --- |
| ENSG00000270661 | 0.0228 | 0.169 |
| ENSG00000256540 | 0.0267 | −0.179 |
| ENSG00000261175 | 0.0375 | −0.117 |
| ENSG00000259054 | 0.0112 | −0.226 |
| ENSG00000245694 | 0.0084 | 0.224 |
