# Supplementary material for: LncRNA RCAT1 promotes tumor progression and metastasis via miR-214-5p/E2F2 axis in renal cell carcinoma
Source: Cell Death Dis. 2021 Jul 9;12(7):689. doi: 10.1038/s41419-021-03955-7 (PMC8270952; doi:10.1038/s41419-021-03955-7)
Supplement: Supplementary file 4 — Table S3. [file 41419_2021_3955_MOESM4_ESM.doc]

**Table S3. The sequence of siRNAs or mimics used in the study.**

| **Target** | **Sequence (5’-3’)** |
| --- | --- |
| LncRNA RCAT1 | GCUUCCAAACCUGGCCAAA |
| E2F2 | CCGUGCUGUUGGCAACUUU |
| miR-214-5p mimics | UGCCUGUCUACACUUGCUGUGC |
