# Supplementary material for: LncRNA RCAT1 promotes tumor progression and metastasis via miR-214-5p/E2F2 axis in renal cell carcinoma
Source: Cell Death Dis. 2021 Jul 9;12(7):689. doi: 10.1038/s41419-021-03955-7 (PMC8270952; doi:10.1038/s41419-021-03955-7)
Supplement: Supplementary file 5 — Table S4. [file 41419_2021_3955_MOESM5_ESM.doc]

**Table S4. Primers used in the study.**

| **Gene** | **Forward (5’-3’)** | **Reverse (5’-3’)** |
| --- | --- | --- |
| LncRNA RCAT1 | CCTGTTCGTACCTCCCTT | TGAAATCTTTGTGATGTGCC |
| E2F2 | AGACTCGGTATGACACTTCGC | GGATGCCTTCCAGCACGT |
| Actin | CATGTACGTTGCTATCCAGGC | CTCCTTAATGTCACGCACGAT |
| U6 | CTCGCTTCGGCAGCACA | AACGCTTCACGAATTTGCGT |
